# Characterisation and manipulation of docetaxel resistant prostate cancer cell lines

**DOI:** 10.1186/1476-4598-10-126

**Published:** 2011-10-07

**Authors:** Amanda J O'Neill, Maria Prencipe, Catherine Dowling, Yue Fan, Laoighse Mulrane, William M Gallagher, Darran O'Connor, Robert O'Connor, Aoife Devery, Claire Corcoran, Sweta Rani, Lorraine O'Driscoll, John M Fitzpatrick, R William G Watson

**Affiliations:** 1 UCD School of Medicine and Medical Science, UCD Conway Institute of Biomolecular and Biomedical Research, University College Dublin, Dublin, Ireland; 2 UCD School of Biomolecular and Biomedical Science, UCD Conway Institute of Biomolecular and Biomedical Research, University College Dublin, Dublin, Ireland; 3 National Institute for Cellular Biotechnology, Dublin City University, Ireland; 4 School of Pharmacy and Pharmaceutical Sciences, Trinity College Dublin, Dublin, Ireland

**Keywords:** Docetaxel, Prostate, NF-κB, Apoptosis, Viability

## Abstract

**Background:**

There is no effective treatment strategy for advanced castration-resistant prostate cancer. Although Docetaxel (Taxotere^®^) represents the most active chemotherapeutic agent it only gives a modest survival advantage with most patients eventually progressing because of inherent or acquired drug resistance. The aims of this study were to further investigate the mechanisms of resistance to Docetaxel. Three Docetaxel resistant sub-lines were generated and confirmed to be resistant to the apoptotic and anti-proliferative effects of increasing concentrations of Docetaxel.

**Results:**

The resistant DU-145 R and 22RV1 R had expression of P-glycoprotein and its inhibition with Elacridar partially and totally reversed the resistant phenotype in the two cell lines respectively, which was not seen in the PC-3 resistant sublines. Resistance was also not mediated in the PC-3 cells by cellular senescence or autophagy but multiple changes in pro- and anti-apoptotic genes and proteins were demonstrated. Even though there were lower basal levels of NF-κB activity in the PC-3 D12 cells compared to the Parental PC-3, docetaxel induced higher NF-κB activity and IκB phosphorylation at 3 and 6 hours with only minor changes in the DU-145 cells. Inhibition of NF-κB with the BAY 11-7082 inhibitor reversed the resistance to Docetaxel.

**Conclusion:**

This study confirms that multiple mechanisms contribute to Docetaxel resistance and the central transcription factor NF-κB plays an immensely important role in determining docetaxel-resistance which may represent an appropriate therapeutic target.

## Background

Unfortunately there is no effective treatment strategy for advanced castration-resistant prostate cancer [1,2]. Although Docetaxel (Taxotere^®^) currently represents the most active chemotherapeutic agent it only gives a modest survival advantage with most patients eventually progressing because of inherent or acquired drug resistance. A number of mechanisms have been proposed to contribute to this resistance. Firstly, the majority of prostate tumours are slow growing even in metastatic disease and thus are unlikely to respond to drugs that are S phase-dependent [3]. Secondly, failure of chemotherapy may be caused by reduced intracellular concentrations of a drug through either increased efflux or decreased intake secondary to alterations in drug transporters, particularly P-glycoprotein (P-gp). Multidrug resistance (MDR) mechanisms including increased expression of the P-gp or increased cellular metabolism of drug detoxifying proteins, such as glutathione-S-transferase, have been shown to protect the cancer cells against cytotoxic drugs [4]. Thirdly, alterations in β-tublin isotypes with different kinetics of microtubule formation have been shown to contribute to resistance. With an increase in isotypes III and IV correlating with Docetaxel resistance *in vitro *[5]. Fourthly, mutations in tumour suppressor proteins, such as loss of PTEN which is a common event occurring in about 60% of prostate cancer patients results in the activation of the phosphatidylinositol 3'-kinase (PI3K) signal transduction cascade resulting in increased cellular proliferation and survival mediated by AKT [6]. Finally, as the prostate cancer phenotype progresses there is the expression of survival factors that inhibits the apoptotic cell death pathway [7], mediated in part by the activation of AKT and other survival signalling pathways. Studies by our group and others have identified elevated protein levels of Bcl-2 [7], Inhibitors of Apoptosis proteins (cIAP-1, cIAP-2, XIAP and Survivin) [8], Clusterin and Heat Shock Proteins [9], as important anti-apoptotic proteins in the development of resistance to a number of apoptotic triggers. However the identification and manipulation of these multiple mechanisms represents a significant challenge as targeting individual proteins has little clinical impact. This was demonstrated in a recent phase II clinical trial with oblimersen sodium, a Bcl-2 antisense oligonucleotide and Docetaxel which did not achieve its primary endpoint of reducing PSA and was associated with increased toxicity [10]. However strategies to block multiple Bcl-2 family members are under way with AT-101, a small molecular inhibitor of Bcl-2, Bcl-xl, Bcl-w and Mcl-1 (clinicaltrials.gov ID: NCT00571675).

Another approach to block multiple downstream genes is to inhibit central transcription factors. There is increasing evidence that inflammation drives the development and progression of prostate cancer [11]. Nuclear factor kappa B (NF-κB) is a central transcription factors activated by inflammation and other cells stresses including paclitaxel [12]. Inhibition of NF-κB with CAPE increases caspase dependent cell death in PC-3 cells mediated via a reduction in IAP expression [12]. Indirect inhibition of NF-κB with an IKK complex inhibitor enhances Docetaxel induced apoptosis in PC-3 and DU-145 cells [13].

This study was undertaken to further investigate the mechanisms of resistance to Docetaxel. A number of Docetaxel resistant sub-lines were generated in the androgen-independent (PC-3, DU-145) and sensitive (22RV1) cell lines. Resistance in the 22RV1 R cells was explained via over expression of P-gp which could be reversed by its inhibition. DU-145 cells have lower levels of P-gp and the resistance was partially blocked by Elacridar. PC-3 cells had no detectable levels of P-gp and Elacridar had no effect on resistance to Docetaxel. Resistance was also not mediated by cellular senescence or autophagy in the PC-3 cells, but multiple changes in pro- and anti-apoptotic genes and proteins were demonstrated. Even though there were lower basal levels of NF-κB activity in the PC-3 D12 cells compared to the Parental PC-3, docetaxel induced higher levels of NF-κB activity and IκB phosphorylation with only minor changes in the DU-145 cells. Inhibition of NF-κB with the BAY 11-7082 inhibitor reversed the resistance to Docetaxel.

## Materials and methods

### Cell culture and resistant cell line development

The human prostate cancer cell lines PC-3, DU-145 and 22RV1 were purchased from the American Type Culture Collection (ATCC) and maintained in RPMI-1640 medium supplemented with 10% Fetal Bovine Serum (FBS), 50 U/ml penicillin/50 μg/ml streptomycin and 2 mM L-glutamine (Invitrogen).

PC-3 resistant sub-lines were generated by initially treating with Docetaxel (Sigma) at 4 nM and 8 nM (suspended in dimethyl sulfoxide (DMSO) (Fluke)) in 75 cm^2 ^flasks for 48 hours. After treatment, the surviving cells were re-seeded into new flasks and allowed to recover for 2-3 weeks. After 5 (at 8 nM) and 7 (at 4 nM) treatments, the dose of docetaxel was increased from 4 nM and 8 nM to 8 nM and 12 nM respectively. The cells underwent a total of 18 treatment cycles at 8 nM and 12 treatment cycles at 12 nM, (cell passage numbers 21-55). Following each treatment they were allowed to fully recover before assessing their resistance to docetaxel and any experimental work. As the passage number of these treated cells increased over time, a subset of PC-3 cells were aged alongside these cells as an appropriate control to ensure that the effects seen were due to resistance rather than due to an ageing effect of the PC-3 cells (PC-3 Ag). In all subsequent experiments, the resistant cells are referred to as PC-3 D8 and PC-3 D12 (reflecting the final treatment doses), and the ageing control cells are referred to as P-C3 Ag. Batches of cells were frozen down and all experiments were carried out on similar passages.

Docetaxel resistant cell lines (DU-145 R; 22RV1 R) were developed over a period of 6 months by stepwise increased concentrations of docetaxel. Cells were continuously maintained in docetaxel, with treatments beginning at the initial IC_50 _of the respective parent cell lines. Media containing docetaxel was changed every 2-3 days. As cells displayed resistance to treatments of docetaxel the concentration was subsequently increased with final treatment doses of 100 nM. Resistance was judged based on decreased cell death and increased proliferation of cells. Age-matched parent cells (DU-145 Ag; 22RV1 Ag) which did not receive treatment were also maintained in culture. Batches of cells were frozen down and all experiments were carried out on similar passages.

### Quantification of apoptosis and viability

Apoptotic events were described as a percentage of total events with hypodiploid DNA assessed by propidium iodide incorporation as previously described [14]. Cells were harvested by trypsinisation, permeabilised with a hypotonic fluorochrome solution (50 mg/ml PI, 3.4 mM sodium citrate, 1 mM Tris, 0.1 mM EDTA, and 0.1% Triton X-100) and incubated on ice for 10 minutes prior to analysis. Samples were run on a Beckman-Coulter FC-500 Cytometer. Ten thousand events were gated on PI intensity and analysed using Mplus software.

#### NF-κB Inhibitor

Cells (100,000 cells/well) were pre-treated with the NF-κB inhibitor, BAY 11-7082 (Merck), (5 μM) for 24 hours after which they were treated with docetaxel (20 nM) for a further 48 hours before been assessed for apoptosis as previously described above.

#### P-glycoprotein Inhibitor

Cells (100,000 cells/well) were pre-treated with the P-glycoprotein inhibitor, Elacridar (DCU), (0.25 & 0.5 μM) for 24 hours after which they were treated with docetaxel (20 nM) for a further 48 hours before been assessed for apoptosis as previously described above.

### 3-(4,5)-dimethylthiazol-2-yl-2,5-diphenyltetrazolium bromide (MTT) assay cell viability assay

Cell viability was assessed by MTT cell staining as previously described [15]. Ten thousand cells/well were cultured in a 96-well plate. Twenty-four hours later, cells were treated with several concentrations of Docetaxel (10 nM, 20 nM, 50 nM and 100 nM) for 24, 48 and 72 hours. MTT (50 μl of a 5 mg/ml in PBS; Sigma-Aldrich) was added to each well and the cells were incubated in a CO_2 _incubator at 37°C for 5 hours. Following media removal, the MTT-formazan formed by metabolically viable cells was dissolved in 200 μl of DMSO (Sigma-Aldrich) and the absorbance was measured in a plate reader at 550 nm.

### Senescence-associated-ß-galactosidase activity

Senescence was assessed by staining cells for β-galactosidase expression as previously described [16]. Briefly, 150,000 cells were seeded in 6 well/plates, 24 hours later cells were treated with several concentrations of Docetaxel for specific times as determined in the experimental design. The cells were then washed twice with PBS, fixed with 2% formaldehyde (Sigma) and 0.2% glutaraldehyde (Sigma-Aldrich) in water for 10 minutes and washed again in 2 × PBS washes. Cells were stained with X-gal staining solution (1 mg/ml X-gal, 40 mmol/l citric acid/sodium phosphate pH 6, 5 mmol/l potassium ferrocyanide, 150 mmol/l NaCl, 2 mmol/l MgCl_2_) for 24 hours in a CO_2 _incubator at 37°C and then rinsed in 2 × washes of PBS and counted using a phase contrast microscope (Olympus CK2). Senescent cells were expressed as a percentage of the total number of cells counted (300 cells/well).

### RNA isolation, cDNA synthesis and Real-time RT-PCR analysis using TaqMan Low Density Arrays

Total RNA was extracted using Trizol reagent (Invitrogen, Paisley, UK) using standard procedures as previously described [17]. The RNA was used to generate cDNA as previously described [17]. PCR amplification of cDNA template was performed in a thermal cycler (Perkin Elmer 7700).

Pre-designed TaqMan probe and primer sets for target genes were chosen from an on-line catalogue (Applied Biosystems). Once selected, the sets were factory-loaded into the 384 wells of TaqMan Low Density Arrays (LDAs). The array format was customized on-line with one replicate per target gene. Expression levels of target genes were normalised to 18S rRNA (Additional file 1 lists the 95 genes chosen for inclusion, and their function). Samples were analyzed using the 7900HT system with a TaqMan LDA Upgrade (Applied Biosystems), according to the manufacturer's instructions. In short, single-stranded cDNA (to final concentration of 100 ng as calculated from starting RNA) was combined with water and TaqMan Universal PCR Master Mix, following by loading 100 μl of each sample per port. Thermal cycling conditions were as follows: 50°C for 2 min, 94°C for 10 min, 97°C for 30 s, and 59.7°C for 1 min. Gene expression values were calculated based on the ΔΔ*C*t method, where one sample was designated the calibrator, through which all other samples were analysed. Briefly, Δ*C*t represents the threshold cycle (*C*t) of the target minus that of 18S rRNA and ΔΔ*C*t represents the Δ*C*t of each target minus that of the calibrator. Relative quantities were determined using the equation 2^-ΔΔ^^*C*^^t^. For the calibrator sample (i.e. normal LCM tissue), the equation is relative quantity = 2^-0^, which is 1; therefore, every other sample is expressed relative to this [18].

### Total cellular protein isolation and western blotting

Whole cell lysates were extracted from treated cells grown to 90% confluence on T75 flasks and 6-well plates. Cells were washed in cold PBS (1100 rpm, 1 min, 4°C in a microcentrifuge) and resuspended in NP-40, Tris 10 mM pH 8.0, 60 mM KCl, 1 mM EDTA pH 8.0, 1.0 mM DTT, 10 μl/ml Protease Inhibitor Cocktail (Sigma P8340)/1 ml of lysis buffer and 10 mM PMSF. Samples were then placed on ice for 10 mins and the cell lysate collected after centrifugation (13000 rpm 5 mins at 4°C). Phosphorylated proteins were isolated using the same protocol above with the addition of 10 μl Phosphatase Inhibitor Cocktail (Sigma P2850)/1 ml of lysis buffer [18].

Total cellular protein was determined by means of the Bradford Assay Protein Detection Kit (Bio-Rad). Equal amounts of protein (50 μg) were subjected to SDS polyacrylamide gel electrophoresis on 8-12% gels before being trans-blotted onto Immobilin P (Millipore) membranes as previously described [14]. Western blotting was performed using antibodies to Bax (1/1000, Upstate Biotechnology), Bcl-xL (1/1000), Bcl-2 (1/500, BD Biosciences), Bid (1/1000, Biosource), Clusterin (1/1000), Id-1 (1/200, Santa Cruz), LC3 (1/1000) P-gp antibody (Mdr-1 D11, 1/200, Santa Cruz), HSP 90 (1/500, StressMarq Biosciences Inc.) and β-Actin (1/5000, Sigma), followed by incubation with the appropriate horseradish peroxidase-conjugated secondary antibodies (Cell Signaling). Autophagy was assessed by western blotting, using an antibody against the autophagy marker LC3 (1/1000, Sigma-Aldrich). As a positive control for the induction of autophagy, cells were starved for 2 hours in Earl's balanced salt solution (EBSS). Bafilomycin A1 (20 nM, Sigma) was used as an inhibitor of autophagy. Signals were detected using ECL™ (Pierce).

### Assessment of NK-κB

#### TransAM™ NF-κB Family Transcription Factor Assay Kit

NF-κB activity in nuclear extracts in the cell lines was determined using the the TransAM™ NF-κB Family Transcription Factor Assay Kit (Active Motif, Belgium). Cells were grown to confluency and nuclear extracts prepared as per the TransAM™ Assay Kit manual. Briefly, nuclear extracts were suspended in TransAM lysis buffer and nuclear proteins (12 μg total protein) were incubated with immobilized oligonucleotides containing the NF-κB consensus DNA-binding site (5'-GGGACTTTCC-3') for 1 hour at room temperature. After washing, 100 μl of one of the diluted NFκB antibodies (1:1000 antibody in 1X Antibody Binding Buffer) was added to each well being used, including blank wells for 1 hour at room temperature. After 3 washes, 100 μl of horseradish-peroxidase-conjugated secondary antibody (1:1000 dilutions) were added to each well for 1 hour at room temperature. The colourimetric substrate was then added after 3 subsequent washes, and the plate was allowed to develop for up to 5 minutes. Following this, the reaction was stopped and absorbance read at 450 nm on a SpectraMax M2 plate reader (Molecular Devices, CA, USA). Relative activation of the NF-κB subunits between the three cell lines could then be plotted, with an increase in absorbance being indicative of increased activation.

### NF-κB Reporter assay

NF-κB transcriptional activity was assessed using a plasmid containing the luciferase reporter gene regulated by five copies of an NF-κB responsive element (Promega). A TK-Renilla luciferase plasmid was used as a transfection efficiency control. Plasmids were co-transfected using GeneJuice^® ^Transfection Reagent (Novagen) following the manufacturer's instructions. After 6 hours, cells were treated with or without 50 nM of docetaxel, for 3, 6 and 24 hours. The luciferase and renilla activities were measured using a Dual-Luciferase^® ^reporter assay (Promega). The NF-κB transcriptional activity was expressed as fold change of Relative Luciferase Units (ratio between luciferase light values divided by the renilla light values), taking the untreated controls as the baseline.

### Statistical analysis

Statistical analysis was carried out using independent samples student t-tests. Results were considered statistically significant where p < 0.05 and results are expressed as mean ± the standard deviation.

## Results

### Effects of Docetaxel on (A) Apoptosis and (B) Proliferation in the Docetaxel resistant sublines

**Figure 1:**
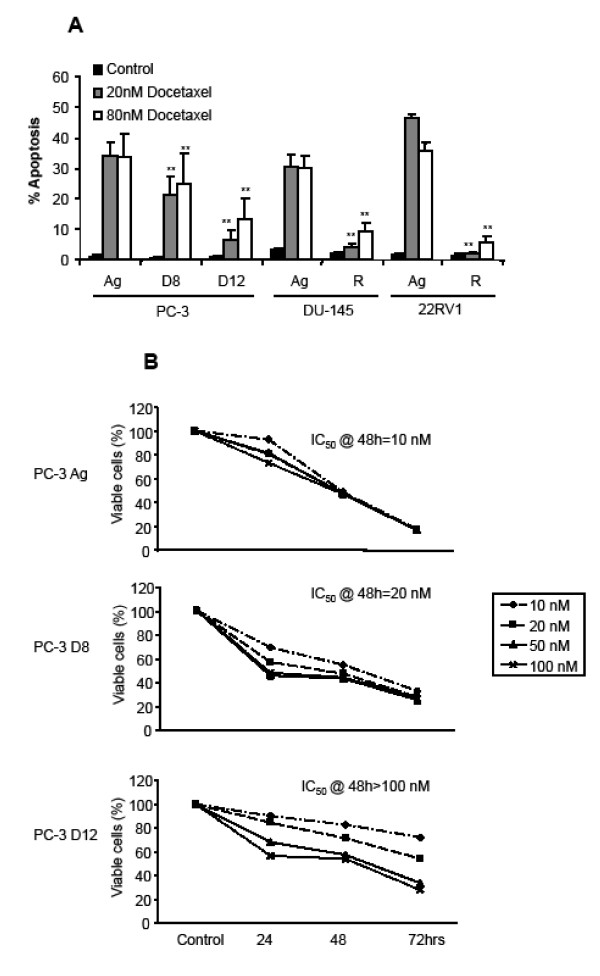
**Effects of Docetaxel on apoptosis and proliferation in the PC-3, DU-145 and 22RV1 Docetaxel resistant sublines**. A. The PC-3 Ag, PC-3 D8, PC-3 D12, DU-145 Ag, DU-145 R, 22RV1 Ag and 22RV1 R cell lines were treated with Docetaxel (20 and 80 nM) for 48 hrs. Apoptosis was assessed by propidium iodide DNA staining and flow cytometry. Values are expressed as mean ± standard deviation. An independent samples t-test was used to compare the mean percentage apoptosis for control, 20 nM and 80 nM for each cell line. **p < 0.001. B. Cell proliferation was measured using the MTT Assay. The PC-3 sublines (PC-3 Ag, PC-3 D8 and PC-3 D12) were treated with increasing doses of Docetaxel (10, 20, 50 & 100 nM) for 24, 48 and 72 hrs. Data shown is representative of three independent experiments.

Figure 1A demonstrates the apoptotic effects of two different concentrations of Docetaxel (20 nM and 80 nM) for 48 hours on the four different Docetaxel resistant sublines. PC-3 D8 and PC-3 D12 demonstrated partial but increasing resistance to docetaxel treatment over the different doses, when compared to the PC-3 Ag. The DU-145 R and 22RV1 R showed significantly more resistance compared to the aged matched control cells (DU-145 Ag & 22RV1 Ag). Cell viability was then determined by the MTT assay (B), following treatment with docetaxel (10 nM, 20 nM, 50 nM and 100 nM) for 24, 48 and 72 hours. The PC-3 Ag cells had an IC_50_@ 48 hrs = 10 nM, the PC-3 D8 an IC_50_@ 48 hrs = 20 nM and the PC-3 D12 an IC_50_@ 48 hrs = 100 nM following treatment with Docetaxel (Figure 1B). This confirmed that the PC-3 D8 and PC-3 D12 sublines had a resistance to Docetaxel treatment.

### P-glycoprotein (P-gp) expression in the Docetaxel resistant sub-lines

**Figure 2:**
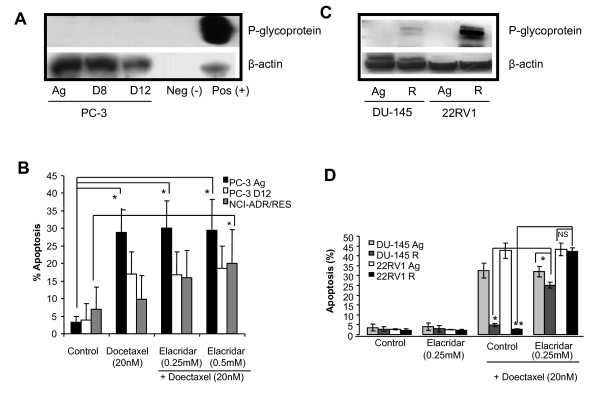
**P-gp expression levels in the Doctaxel resistant cell lines**. A. Total cellular protein was extracted from the cells and 50 μg was assessed by western blotting. P-gp was not expressed in the PC-3 Ag, PC-3 D8, PC-3 D12 sublines or the DLKP (40 μg) negative control cell line, but was expressed in the DLKP-A (5 μg) drug resistant variant of the DLKP cell line. β-actin the house keeping protein was used as loading control for P-Gp. B. The PC-3 Ag, PC-3 D12 and NCI-ADR/RES cells lines were treated with Docetaxel (20 nM) alone or in the presence of the P-gylcoprotein inhibitor Elacridar (0.25 and 0.5 μM) and Docetaxel (20 nM) and assessed for apoptosis by flow cytometry. An independent samples t-test was used to compare the average percentage of apoptosis between the control groups and each of the treated groups for the PC-3 Ag, PC-3 D12 and NCI-ADR/RES cell lines *p- < 0.05. C. Total cellular protein was extracted from the cells and 50 μg was assessed by western blotting. P-Gp was weakly expressed in the DU-145 R and strongly expressed in the 22RV1 R sublines. No expression was seen in the Aged matched. β-actin the house keeping protein was used as loading control for p-glycoprotein. D. The DU-145 Ag, DU-145 R and 22RV1 Ag and 22RV1 R cells lines were treated with Docetaxel (20 nM) alone, or the P-Gp inhibitor Elacridar (0.25 μM) alone or in the presence of Elacridar (0.25 μM) and Docetaxel (20 nM) and assessed for apoptosis by flow cytometry. Blots shown are representative of three independent experiments.

We next wanted to investigate the mechanisms responsible for Docetaxel resistance. We firstly examined the expression of the classical drug pump, P-gp (Mdr-1) in the PC-3 D8 and PC-3 D12 sublines compared to the PC-3 Ag subline. Figure 2A clearly shows no expression of P-gp in any of the PC-3 sublines, when compared to the P-gp positive cell line (DLKP-A, Figure 2A), and the DLKP negative cell line, as previously described [19].

We further confirmed P-gp was not playing a role in this resistance by blocking P-gp activity with the P-gp inhibitor, Elacridar (0.25 and 0.5 μM) [20,21]. Following 24 hours, pre-treatment, Elacridar had no effect on the cells susceptibility to Docetaxel (20 nM) induced apoptosis over 48 hours. However, as a positive control the P-gp over expressing cell line NCI/ADR/RES (ovarian cancer cell line) underwent increased levels of apoptosis following treatment with Docetaxel (20 nM) after 48 hours (Figure 2B).

Similar experiments were carried out with the DU-145 R and 22RV1 R sublines. Western blotting demonstrated expression of P-gp in the DU-145 R and 22RV1 R sublines with higher expression in the 22RV1 R (Figure 2C). Elacridar treatment (0.25 and 0.5 μM) also partially reversed the resistance to apoptosis in the DU-145 R cells and totally reversed the resistance in the 22RV1 R sublines following treatment with Docetaxel (20 nM) for 48 hours (Figure 2D). As the resistance to Docetaxel induced apoptosis may be partially explained by the over expression of P-gp in the DU-145 R and 22RV1 R cells we focused on the PC-3 D8 and PC-3 D12 sublines whose resistance was not P-gp dependent.

### Cellular senescence and autophagy as mechanisms of Docetaxel resistance

**Figure 3:**
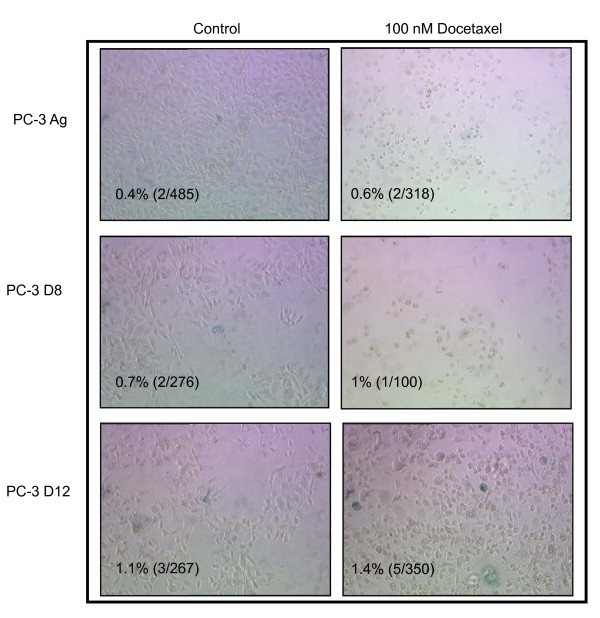
**Cellular sensecence in response to Docetaxel treatment**. The PC-3 Ag, PC-3 D8 and PC-3 D12 cells were plated in 6 well plates at 300,000/well and grown for 24 hours. The cells were then treated with Docetaxel (100 nM) for 48 hours and fixed with 2% formaldehyde and 0.2% glutaraldehyde. Senescent cells were then assessed by staining cells for β-galactosidase expression. Positive cells were counted and expressed as a percentage of the total number of cells in the well. Pictures are a representation of 5 independent experiments.

Senescent cells demonstrate resistance to apoptosis and express β-galactosidase. The effects of Docetaxel treatment (100 nM for 48 hours) on PC-3 Ag, PC-3 D8 and PC-3 D12 cellular senescence is demonstrated in Figure 3 which shows no significant increase in β-galactosidase staining.

**Figure 4:**
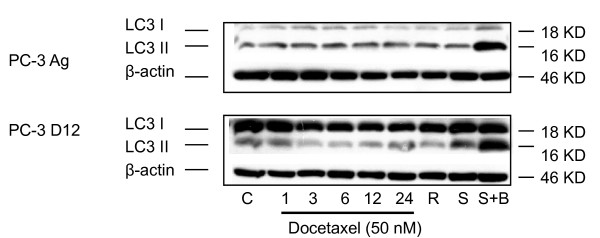
**Autophagy as a mechanisms of Docetaxel resistance**. Following treatment of the Aged matched controls (Upper panel) and PC-3 D12 resistant sublines (Bottom panel) with 50 nM of Docetaxel for 1, 3, 6, 12 and 24 hrs, total cellular protein was extracted and assessed for LC3II by western blotting using the relevant anti-body and conditions as described in the methods section. For positive controls cells were either treated with Rapamycin (R, 0.2 μM) for 4 hrs, Starvation (S, the cells were starved in EBSS (Earls Balanced Salt Solution) medium for 2 hrs), or Starvation plus Bafilomycin (S+B, 20 nM) for 2 hrs. β-actin the house keeping protein was used as loading control. Blots shown are representative of three independent experiments.

Autophagy (macroautophagy) is also a well conserved mechanism by which cells adapt to stress such as starvation [22]. This complex cellular process has recently been associated with resistance to cancer therapies [23]. Total cellular protein was extracted from the different cell lines following Docetaxel treatment (50 nM) and assessed for the expression of LC3 II, a protein associated with autophagosome formation and a marker of autophagy [24]. While the resistant PC-3 D12 sub-lines showed a higher baseline expression of LC3I, the precursor of LC3II, no consistent difference in the baseline expression levels of LC3II was demonstrated between the PC-3 Ag and the resistant PC-3 D12 subline (Figure 4) following treatment.

### Altered expression of apoptotic related genes in the Docetaxel resistant sublines

Having ruled out P-gp, senescence and autophagy as possible mechanisms of resistance in the PC-3 resistant sublines we next investigated other mechanisms and focused on the alteration in genes and proteins which regulate cellular apoptosis. Custom designed Low Density Arrays (LDA) which included the probes for the IAPs, death receptors, death ligands, and signalling molecules as well as genes involved in cell cycle regulation, DNA damage and repair and chemotherapy resistance were developed. Additional file 1, lists the 95 genes chosen for inclusion and their function.

Table 1 demonstrates the genes which were increased in the PC-3 D8 subline compared to the PC-3 Ag cells. The majority of these genes are anti-apoptotic including BIRC7 (Livin-a member of the IAP family), Bcl2-A1 (Bcl-2 member), Foxo1A and HSP 90. Clusterin, a molecular chaperone was also found to be increased. TNF receptor family member 10C, which is known to inhibit TRAIL-induced apoptosis, was also increased in this subline. Nibrin and p73 which are involved in DNA damage and repair were also up-regulated. Table 2 demonstrates the genes which were down-regulated in the PC-3 D8 subline compared to the PC-3 Ag cells. A number of genes involved in the induction of apoptosis were shown to be changed. These include; FOXO1, NGFR, TRAF-1, and TRAF-2. Surprisingly a number of anti-apoptotic genes were also decreased including; MCL-1 and BIRC3.

**Table 1:** Genes increased in the PC-3 D8 Docetaxel Resistant Cell line

PC-3 D8 vs PC-3 Ag Gene Name	Accession no.	Fold change	p value
BIK	NM_001197	4.606724005	0.024378
FOXO1	NM_002015	4.092077589	0.006372
NAIP	NM_004536	3.895819042	NA
HSP90AA1	NM_001017963	2.255183574	0.116769
IL8	NM_000584	2.194814925	0.060076
BCL2A1	NM_001114735	2.003718027	0.018472
NBN	NM_002485	1.994366021	0.011912
TNFRSF10C	NM_003841	1.931533233	0.288353
TP73	NM_001126240	1.785086313	0.082871
BNIP3L	NM_004331	1.747205486	0.03454
CLU	NM_001171138	1.740002897	0.304795
APAF1	NM_001160	1.673345235	0.38724
FASLG	NM_000639	1.563978305	0.73945
BNIP3	NM_004052	1.50139036	0.173556

Pre-designed TaqMan probe and primer sets for target genes were chosen from an on-line catalogue (Applied Biosystems). Expression levels of target genes were normalised to concentration of 18S rRNA (Additional File 1 lists the 95 genes chosen for inclusion and their function). Total mRNA was extracted from the PC-3 Ag and PC-3 D8 cells and used on custom designed Low Density Arrays (LDA) to determine which genes were increased (fold increase > 1.5) in the Docetaxel resistanct PC-3 D8 subline compared to the PC-3 Ag. n = 3.

**Table 2:** Genes decreased in the PC-3 D8 Docetaxel Resistant Cell line

PC-3 D8 vs PC-3 Ag Gene Name	Accession no.	Fold change	p value
BAG1	NM_001172415	0.485055	0.102992
MCL1	NM_021960	0.464403	0.048121
ID1	NM_002165	0.460689	0.434385
ATR	NM_001184	0.435832	0.039411
PTEN	NM_000314	0.422497	0.245383
NGFR	NM_002507	0.391286	0.285516
BNIP2	NM_004330	0.36245	0.124787
JUN	NM_002228	0.339603	0.18277
BIRC3	NM_001165	0.313233	0.006408
BMF	NM_001003940	0.292831	0.104867
DDIT3	NM_001195053	0.279562	0.157249
TRAF1	NM_001190945	0.269089	0.18055
IL6	NM_000600	0.190685	0.031992
IGF1R	NM_000875	0.160304	0.013039
MAPK10	NM_002753	0.123932	NA
TNFSF10	NM_001190942	0.115113	0.175825
BIRC8	NM_033341	0.024782	0.517705
TNFRSF10D	NM_003840	0.011276	0.144385

Genes whose expression has decreased (fold increase <0.5) in the Docetaxel resistant PC-3 D8 subline compared to the PC-3 Ag. n = 3.

Genes increased in the PC3-D12 subline compared to the PC-3 Ag cells are represented in Table 3. Many of the increased genes are anti-apoptotic genes such as BIRC1, BCL2-A1, FOXO1A, NOL3 and Clusterin. Genes involved in DNA damage and repair such as Nibrin, Chek-1 and ATM were also increased. Decreased gene expression in the PC3-D12 subline compared to the PC-3 Ag cells is demonstrated in Table 4. Many of these genes are those involved in the induction of apoptosis. These include BOK, NGFR, Fas, FasLG, TNF receptor member 11b, TRAF-1 and TRAIL. A number of genes involved in cell cycle regulation and DNA damage detection were also decreased. These include Chek2, p21, ETS2 and ATR.

**Table 3:** Genes increased in the PC-3 D12 Docetaxel Resistant Cell line

PC-3 D12 vs PC-3 Ag Gene Name	Accession no.	Fold change	p value
NAIP	NM_004536	28.14023	NA
BCL2A1	NM_001114735	9.956213	7.84E-06
CLU	NM_001171138	6.138126	0.019757
TNFRSF1B	NM_001066	4.568718	NA
ATM	NM_000051	2.884063	0.013665
FOXO1	NM_002015	2.746056	0.0167
MAP2K4	NM_003010	2.456245	0.13486
APAF1	NM_001160	2.264102	0.34641
NOL3	NM_001185057	1.946074	0.101148
CHEK1	NM_001114121	1.554884	0.433367
BNIP3L	NM_004331	1.514918	0.113779
NBN	NM_002485	1.505995	0.051555

Total mRNA was extracted from the PC-3 Ag and PC-3 D12 cells and used on custom designed Low Density Arrays (LDA) which included the transcriptional profile probes for the IAPs, death receptors, death ligands, and signalling molecules as well as genes involved in cell cycle regulation, DNA damage and repair and chemotherapy resistance. Additional File 1, lists the 95 genes chosen for inclusion, and their function. Genes whose expression has increased (fold increase > 1.5) in the Docetaxel resistanct PC-3 D12 subline compared to the PC-3 Ag. n = 3.

**Table 4:** Genes decreased in the PC-3 D12 Docetaxel Resistant Cell line

PC-3 D12 vs PC-3 Ag Gene Name	Accession no.	Fold change	p value
XIAP	NM_001167	0.481312	0.176898
CHEK2	NM_001005735	0.475224	0.131629
JUN	NM_002228	0.46633	0.309774
BCL2L2	NM_004050	0.45255	0.082823
TRAF2	NM_021138	0.443326	0.006969
DDIT3	NM_001195053	0.432559	0.452576
BCL2L11	NM_006538	0.432054	0.074605
CDKN1A	NM_000389	0.42579	0.080132
ETS2	NM_005239	0.382415	0.00487
IL8	NM_000584	0.369938	0.022567
FAS	NM_000043	0.336752	3.61E-05
ATR	NM_001184	0.315906	0.010488
BOK	NM_032515	0.298213	0.014457
NGFR	NM_002507	0.289875	0.003775
ABCB1	NM_000927	0.254066	NA
FASLG	NM_000639	0.251687	0.330804
BIK	NM_001197	0.249128	0.04058
XAF1	NM_017523	0.196716	0.637745
VEGFA	NM_001025366	0.147831	0.017993
TRAF1	NM_001190945	0.13621	0.030832
BIRC3	NM_001165	0.102653	0.000293
TNFRSF11B	NM_002546	0.099691	0.005951
TNFRSF10D	NM_003840	0.085696	0.332193
EGR1	NM_001964	0.0682	0.005374
TP73	NM_001126240	0.026123	0.004742
BIRC8	NM_033341	0.024998	0.523298
TNFSF10	NM_001190942	0.01728	0.053714
IL6	NM_000600	0.011894	0.000652

Genes whose expression has decreased (fold increase <0.5) in the Docetaxel resistanct PC-3 D12 subline compared to the PC-3 Ag. n = 3.

### Validation of altered gene expression

**Figure 5:**
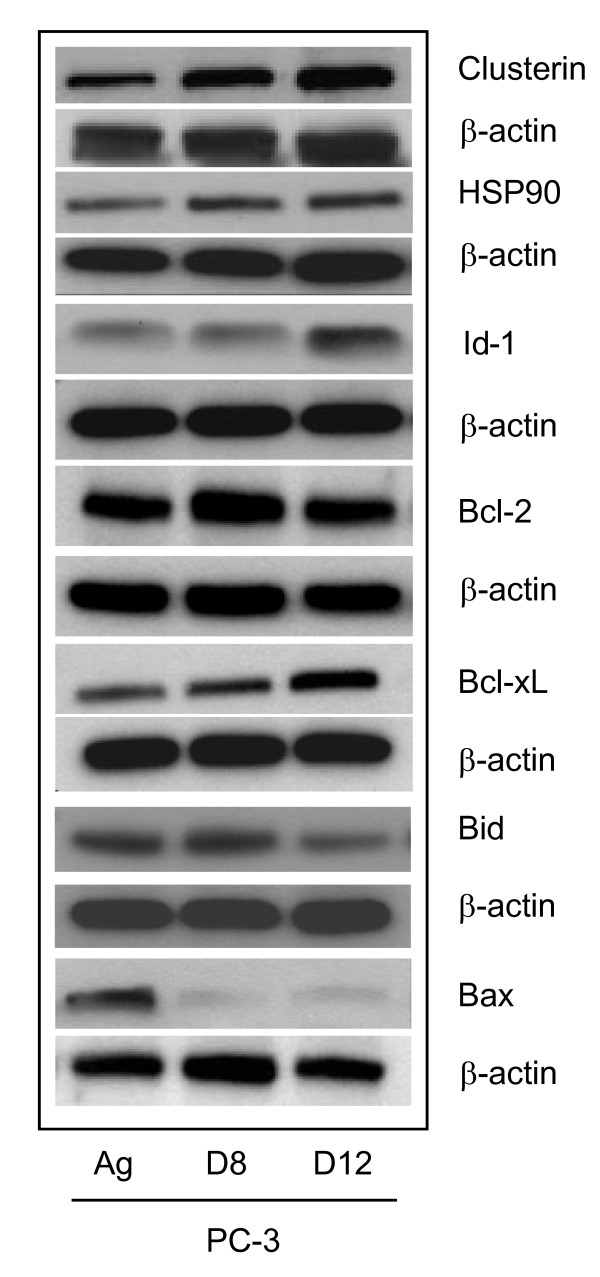
**Validation of Low Density Arrays**. Total cellular protein was extracted from the PC-3 sublines (PC-3 Ag, PC-3 D8 & PC-3 D12) and 50 μg was loaded onto a western blot to assess the expression of Clusterin, HSP90, Id-1, Bcl-2, Bcl-xL, Bax, and Bid using their respective antibodies as described in the Method section. The house keeping protein β-actin was used as a loading control. Blots shown are representative of three independent experiments.

We next wanted to validate specific genes at the protein level. Total cellular protein was extracted from the PC-3 Ag, PC-3 D8 and PC-3 D12 and assessed by western blotting for Clusterin, Id-1, Bcl-2, Bcl-xL, Bid and Bax. As demonstrated in Figure 5, Id-1 expression was increased in the PC-3 D12 subline only, while the protein expression of Bcl-2 was increased in PC-3 D8 but not in PC-3 D12. Anti-apoptotic Bcl-xL was increased in both sublines. Pro-apoptotic protein Bid was decreased in PC-3 D12 while Bax was decreased in both sublines. Clusterin was significantly increased in both sublines. The molecular chaperone HSP90 was also increased in both sublines (Figure 5).

### Docetaxel treatment increases NF-κB activity in the PC-3 D12 resistant PC-3 subline

**Figure 6:**
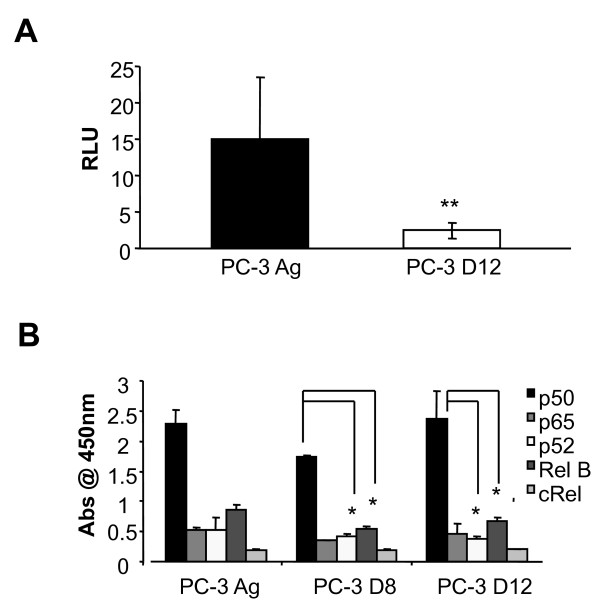
**NF-κB transcriptional activity and Baseline NFkB subunit activity**. A. PC-3 Ag and PC-3 D12 cells were plated at 100,000/per well and grown for 24 hours. The baseline NFκB activity was assessed by luciferase assay, using a Dual Luciferase Assay from Promega following the manufactures instructions. Averages from 3 independent experiments are shown. Mean values were compared using t-test assuming equal variances. B. The PC-3 Ag and PC-3 D12 cells were grown to confluency and nuclear extracts prepared as per the TransAM™ Assay Kit manual. Averages from 3 independent experiments are shown. Mean values were compared using t-test assuming equal variances.

As NF-κB regulated the expression of a number of the apoptotic genes listed in Tables 1, 2, 3 and 4, we next wanted to determine whether NF-κB played a role in the resistance to Docetaxel. We firstly assessed the baseline transcriptional activity in the PC-3 resistant subline using a luciferase assay. Interestingly, NF-κB transcriptional activity was significantly decreased (p < 0.001) in the resistant PC-3 D12 subline compared to the PC-3 Ag cells (Figure 6A). The DU145 cell lines showed a similar trend with decreased NF-κB transcriptional activity in the resistant subline (DU-145 R) compared to the parental (DU-145 Ag); while there was no difference in NF-κB activity in the aged (22RV1 Ag) matched and resistant 22RV1 (22RV1 R), being the baseline NF-κB activity in these cells was very low in comparison to the PC-3 and DU145 cells (data not shown). NF-κB decreased activity in the resistant PC-3 sublines was confirmed by the TransAM ELISA based assay which showed a significant decrease in the activity of p52 (p < 0.01) and RelB (p < 0.05) subunits, in both the PC-3 D8 and PC-3 D12 resistant cells compared to the PC-3 Ag (Figure 6B).

**Figure 7:**
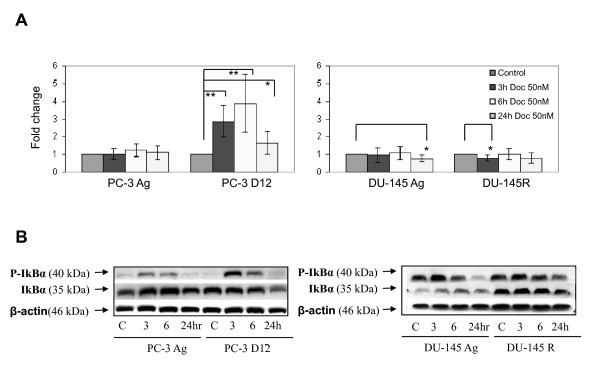
**NF-κB transcriptional activity following docetaxel treatment**. A. The PC-3 and DU-145 aged matched and Docetaxel resistant sublines were treated with 50 uM concentrations of Docetaxel for 3, 6 and 24 hours at which time NFκB activity was assessed by luciferase assay, using a Dual Luciferase Assay from Promega following the manufactures instructions. Graphs are generated from 3 independent experiments. Mean values were compared using t-test assuming equal variances. * p < 0.01; ** p < 0.0001. B. Cells were treated as above and at the different time points total cellular protein was extracted and 50 ug loaded onto western blots and assessed for P-IkBα and IkBα. β-actin was used as an additional loading control. Blots are representative of three independent experiments.

**Figure 8:**
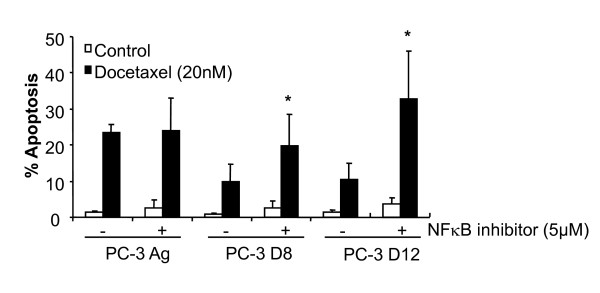
**NF-κB inhibitor studies following docetaxel treatment**. The PC-3 sublines where pre-treated with the NFκB inhibitor (BAY 11-7082, 5 μM) for 24 hours followed by Docetaxel (20 nM) for a further 48 hours and assessed for apoptosis by flow cytometry. Graphs represent the results of 3 independent experiments. Mean values were compared using t-test assuming equal variances. * p < 0.05.

We then assessed the effects of Docetaxel treatment on NF-κB transcriptional activity in the resistant subline. Following Docetaxel treatment there was significantly increased transcriptional activity in the PC-3 D12 resistant subline compared to the PC-3 Ag cells (Figure 7A). There was no increase in the DU-145 R subline (Figure 7A), and there was no NF-κB activity in the 22RV1 cells neither in response to docetaxel or to the classical NF-κB inducers such as TNFα or LPS (data not shown). Activation of NF-κB in the resistant PC-3 D12 subline compared to the PC-3 Ag cells was confirmed by western blotting showing higher levels of phospho-IκBα in the PC-3 D12 compared to the PC-3 Ag, following 3-6 h of docetaxel treatment (Figure 7B). NF-κB was functionally confirmed to be centrally involved in resistance to Docetaxel using the NF-κB inhibitor (BAY 11-7082, Merck) which reversed resistance in both the PC-3 D8 and PC-3 D12 sublines (Figure 8).

## Discussion

The treatment of advanced prostate cancer represents a significant challenge due to the accumulation of multiple mechanisms of drug resistance. Resistance to Docetaxel treatment includes changes in classical multiple drug resistant pathways, expression of different β-tublin isotypes, mutations in tumour suppressor proteins and altered expression of pro- and anti-apoptotic proteins [25]. We have generated three models of docetaxel-resistance in prostate cancer cells which may mimic the situation in the clinical setting and used them to understand the complexity of mechanisms leading to treatment resistance. Understanding these pathways will be essential if we want to better impact on the outcome of these patients and identify additional co-treatments which will manipulate this resistance and develop personalised treatments. We demonstrated that all four sublines are resistant to increasing concentrations of Docetaxel as assessed by apoptosis and viability assays.

One of the main mechanisms of resistance to Docetaxel is the over expression of P-gp [4] which would reduce intracellular concentrations of the drug through increased efflux. Chemoresistant PC-3 cells do not over express P-gp, while resistant DU145 cells exhibit both over expression of the protein and restored chemosensitivity with P-gp inhibition [5,26]. This has been the basis for the development of cabazitaxel which can overcome P-gp over expression [27] and is now used as a second line therapy for patients who fail Docetaxel treatments. In our study we demonstrated that the resistance in the PC-3 D8 and PC-3 D12 sublines was independent of P-gp over expression as there were no detectable levels and no effects of the P-gp inhibitor Elacridar on their resistance. However the DU-145 and 22RV1 cells expressed increasing levels of P-gp and Elacridar totally reversed the resistance in the 22RV1 and partially in the DU145 resistant sublines. Based on these results we focused on the PC-3 cells to understand the mechanisms of resistance independent of P-gp.

We next investigated cellular senescence as a possible mechanism of resistance. Premature cellular senescence, a phenomenon by which cells stop dividing and acquire a typical morphology and an altered gene expression profile in response to stress [3], has recently been associated with chemotherapy resistance. We performed a ß-galactosidase assay to assess cellular senescence following docetaxel treatment. Although ß-galactosidase staining showed a modest increase in the percentage of senescent cells following docetaxel treatment, this increase was considered to be insufficient to explain the resistance of the PC-3 D8 and PC-3 D12 sublines to docetaxel.

Autophagy (macroautophagy), a well conserved mechanism by which cells adapt to stress such as starvation, has also been recently associated with resistance to cancer therapies [23]. Therefore we investigated whether increased autophagy was involved in the resistance to docetaxel in the PC-3 D12 subline. Although western blotting showed increased expression of the autophagic marker LC3II in the PC-3 D12 compared to the PC-3 Ag cells following docetaxel treatment, this was only a modest effect. Interestingly, the resistant PC-3 D12 subline showed a higher baseline expression of LC3I, the precursor of LC3II, which warrants further investigation.

We next undertook to assess alterations in the apoptotic phenotype of the resistant cells in order to understand the mechanisms involved. Custom-designed LDA containing real-time PCR assays for all the key apoptotic genes including the IAPs, death receptors, death ligands, and signalling molecules as well as genes involved in cell cycle regulation, DNA damage and repair were developed. The LDA results showed a complex interplay between changes in the expression of both pro- and anti-apoptotic proteins. From our study, genes which were shown to be altered in both the docetaxel-resistant sublines (PC-3 D8 and PC-3 D12) included; FOXO1, NGFR, TRAF-1, Mcl-1, BIRC3, BIRC1, Bcl-2-A1, NOL3, Clusterin, BOK, NGFR, Fas, FasLG, TNF receptor member 11b and TRAIL.

Trougakos *et al *demonstrated that secreted clusterin (sCLU) knockdown in human prostate cancer cells induces significant reduction of cellular growth and higher rates of spontaneous endogenous apoptosis [28]. sCLU is a cytoprotective chaperone that stabilizes conformations of proteins during times of cellular stress, thereby inhibiting protein aggregation and precipitation [29]. Additionally, sCLU can interact with and inhibit activated Bax, thereby inhibiting cytochrome C release and caspase activation [30]. In prostate cancer, sCLU levels have previously been correlated with Gleason grade [31]. A recent publication identified sCLU to be over-expressed in a docetaxel-resistant PC-3 subline and reported that knockdown of sClu chemosensitizes this cell line to taxane and mitoxantrone based chemotherapy both *in vitro *and *in vivo *[32].

Bcl-2 related protein A1 (Bcl2-A1) or BLF1 is a member of the Bcl-2 family. Although it is regulated differently from Bcl-2, it has similar anti-apoptotic activity. A recent publication demonstrated that Bcl2-A1 mediates resistance to doxorubicin in haematopoietic cell lines [33].

Modur *et al *found that FOXO1A was highly expressed in normal prostate. They also noted that in PTEN-deficient prostate carcinoma cell lines, FOXO1A was cytoplasmically sequestered and inactive and expression of TRAIL, a pro-apoptotic effector, was decreased. They determined that TRAIL is a direct target of FOXO1A, and they hypothesized that the loss of PTEN contributes to increased tumour cell survival through decreased transcriptional activity of FOXO1A and FOXO3A followed by decreased TRAIL expression and apoptosis [34].

BNIP3L which is also elevated in both sublines, plays a pro-survival role in apoptosis. Yasuda *et al *showed that *in vitro *translated BNIP3L binds specifically and directly with GST fusion proteins of various Bcl-2 family anti-apoptosis proteins, suggesting that, like BNIP3, BNIP3L may antagonize the activity of Bcl-2 family anti-apoptosis proteins [35]. In this setting, BNIP3L may be increased in our resistant sublines in an attempt to antagonize the very elevated levels of Bcl2-A1.

Genes down-regulated in both sublines include TRAIL, NGFR, TNF member 10D and ATR among others (see Tables 1, 2, 3 and 4 results). As PC-3 cells are highly resistant to TRAIL, it is unsurprising that levels of pro-apoptotic TRAIL are further decreased in the resistant sublines. Tumour necrosis factor superfamily member 10D (TNF member 10D) has been shown to act as a decoy receptor for TRAIL (TRAIL R4). Transient over-expression of TRAIL R4 in cells normally sensitive to TRAIL-mediated apoptosis conferred complete protection, and Degli-Esposti *et al *suggested that one function of TRAIL R4 may be the inhibition of TRAIL cytotoxicity [36]. As a large number of genes were either increased or decreased in the docetaxel-resistant sublines, targeting these individually may not lead to alterations in the resistant phenotype but understanding the central signalling pathways and transcription factors would represent a more appropriate therapeutic targeting approach.

NF-κB initiates the transcription of a wide variety of genes that code for angiogenic factors, cell adhesion molecules, anti-apoptotic factors, and cytokines, which are involved in cell survival, invasion, metastasis, and chemoresistance [37]. In addition to its role in oncogenesis, NF-κB has been associated with chemoresistance in various models [38-40]. Recent focus on inhibition of NF-κB activity has identified a sensitisation to apoptosis induced by various apoptotic triggers, including Docetaxel in multiple cell lines including PC-3 cells [41]. Our low density array studies identified the up-regulation of a number of target genes of NF-κB (IAPs, IL-8, TNF receptor family members and FasLG). Based on these findings we focused our investigations towards the central role of NF-κB in mediating the resistant phenotype.

Despite a decrease in the baseline expression levels of the NF-κB subunits p52 and RelB and transcriptional activity, when the cells were treated with Docetaxel there was an increase in NF-κB transcriptional activity. This led us to the inhibition of NF-κB activity using the NF-κB inhibitor BAY11-7082 prior to treatment with Docetaxel which resulted in significant reversal of Docetaxel resistance.

## Conclusion

A mounting body of evidence implicates dysregulation of cell-survival signalling pathways in the pathogenesis of castration-resistant prostate cancer. What is not clear is which are the most salient targets in these pathways that when inhibited will lead to reversal of resistance and the most effective clinical outcome. An improved understanding of the molecular mechanisms regulating castration-resistant prostate cancer will drive both the identification of appropriate treatment strategies and biomarkers to inform such strategies. Our current results indicate that NF-κB plays an important role in determining Docetaxel resistance and attempts to sensitise resistant prostate cancer cells to Docetaxel therapy should target this transcription factor.

## Abbreviations

MDR: multi drug resistance; P-gp: p-glycoprotein; PI3K: phosphatidylinositol 3'-kinase

## Competing interests

The authors declare that they have no competing interests.

## Authors' contributions

AJON undertook the low density arrays and western blotting validation assays as well as preparing and writing the manuscript. MP undertook the NF-κB experiments as well as preparing and writing the manuscript. CD made the Docetaxel resitant PC-3 cell lines and performed the viability and apoptosis experiments. AJON, MP and CD contributed equally to the delivery of the manuscript. YF did the statistical analysis for the manuscript. LM, WMG, and DOC undertook the NFκB Trans Am kit experiments. ROC and AD examined the P-gp protein expression in the cell lines and provided the inhibitor. CC, SR and LOD made the DU-145 and 22RV1 Docetaxel resistant cell lines. JMF and RWGW conceived the study. RWGW was central in the design, coordination and manuscript editing. All authors have read and approved the final manuscript.

## Supplementary Material

Additional file 1 **List of genes on the Low Density Arrays**. This table contains the list of 95 genes contained on the Low Density Arrays including gene name, gene symbol and gene function for each gene included on the array. Click here for file
