# Supplementary material for: Characterisation and manipulation of docetaxel resistant prostate cancer cell lines
Source: Mol Cancer. 2011 Oct 7;10:126. doi: 10.1186/1476-4598-10-126 (PMC3203088; doi:10.1186/1476-4598-10-126)
Supplement: Additional file 1 — List of genes on the Low Density Arrays. This table contains the list of 95 genes contained on the Low Density Arrays including gene name, gene symbol and gene function for each gene included on the array. [file 1476-4598-10-126-S1.DOC]

# Additional files

### Additional file 1

### List of genes on the Low Density Arrays

This table contains the list of 95 genes contained on the Low Density Arrays including gene name, gene symbol and gene function for each gene included on the array.

|  | **Gene Symbol** | **Gene Name** | **Gene Function** | |  |
| --- | --- | --- | --- | --- | --- |
| 1 | ABCB1 | MDR-1 | Glycoprotein involved in multidrug resistance | | |
| 2 | ACTB | β-actin | Modulates cell migration | | |
| 3 | AKT-1 | AKT-1 | Regulates PIK3-dependent survival | | |
| 4 | AMACR | AMACR | Fatty acid metabolism | | |
| 5 | APAF-1 | APAF-1 | Activates pro-caspase 9 | | |
| 6 | AR | Androgen receptor | Function as a coactivator for androgen-dependent transcription | | |
| 7 | ATM | ATM | DNA repair and cell cycle | | |
| 8 | ATR | ATR | DNA repair and cell cycle | | |
| 9 | BAD | BAD | Pro-apoptotic |  | |
| 10 | BAG-1 | Bcl-2associated gene 1 | Inhibitor of apoptosis | | |
| 11 | BAK-1 | BAK-1 | Induction of apoptosis | | |
| 12 | BAX | BAX | Pro-apoptotic | |  |
| 13 | BBC-3 | PUMA | Induction of apoptosis | | |
| 14 | BCL-2 | Bcl-2 | Inhibitor of apoptosis | | |
| 15 | BCL-2A1 | Bc2 related protein A | Inhibitor of apoptosis | | |
| 16 | BCL2L1 | Bcl-xL | Inhibitor of apoptosis | | |
| 17 | BCL2L11 | Bim | Induction of apoptosis | | |
| 18 | BCL2L2 | Bcl-2 like 2 | Inhibitor of apoptosis | | |
| 19 | BID | Bid | Pro-apoptotic | |  |
| 20 | BIK | Bcl-2 interacting killer | Induction of apoptosis | | |
| 21 | BIRC1 | IAP member 1 | Inhibitor of apoptosis | | |
| 22 | BIRC2 | cIAP-1 | Inhibitor of apoptosis | | |
| 23 | BIRC3 | cIAp-2 | Inhibitor of apoptosis | | |
| 24 | BIRC4 | xIAP | Inhibitor of apoptosis | | |
| 25 | BIRC4BP | xIAP-associated factor 1 | Inhibitor of apoptosis | | |
| 26 | BIRC5EPR1 | Survivin (IAP) | Inhibitor of apoptosis | | |
| 27 | BIRC6 | Bruce (IAP) | Inhibitor of apoptosis | | |
| 28 | BIRC7 | Livin (IAP) | Inhibitor of apoptosis | | |
| 29 | BIRC8 | IAP member 8 | Inhibitor of apoptosis | | |
| 30 | BMF | Bcl-2 modifying factor | Induction of apoptosis | | |
| 31 | BNIP1 | Bcl-2-interacting protein 1 | Pro-apoptotic | |  |
| 32 | BNIP2 | Bcl-2-interacting protein 2 | Pro-apoptotic | |  |
| 33 | BNIP3 | Bcl-2-interacting protein 3 | Pro-apoptotic | |  |
| 34 | BNIP3L | Bcl-2-interacting protein 3L | Pro-apoptotic | |  |
| 35 | BOK | Bcl-2 ovarian killer | Pro-apoptotic | |  |
| 36 | CDKN1A | p21 | Cell cycle control and response to DNA damage | | |
| 37 | CFKAR | cFLIP | Regulation of NFκB activity | | |
| 38 | CHEK1 | Chk 1 | DNA damage checkpoint | | |
| 39 | CHEK2 | Chk 2 | DNA damage checkpoint | | |
| 40 | CLU | Clusterin | Cell survival | |  |
| 41 | DDIT3 | DNA damage inducible transcript | Regulation of DNA damage | | |
| 42 | DIABLO | Smac/Diablo | Induction of apoptosis | | |
| 43 | EGR1 | Early growth factor response 1 | Regulation of transcription | | |
| 44 | ETS2 | ETS 2 | Cell cycle control | | |
| 45 | FADD | Fass associated death domain | Mediates signalling from TNF family members | | |
| 46 | FAS | Fas associated death domain | Interacts with Fas L to induce apoptosis | | |
| 47 | FAS LG | Fas Ligand | Induction of apoptosis and regulation of NFκB activity | | |
| 48 | FOXO1A | Forkhead 1A | Anti-apoptotic | |  |
| 49 | FOXO3A | Forkhead 3A | Anti-apoptotic | |  |
| 50 | GAPDH | Gapdh | Involved in carbohydrate metabolism | | |
| 51 | HDAC1 | Histone deacetylase 1 | Cell cycle control | | |
| 52 | HSP90AA1 | HSP 90 | Critical for tumour invasiveness | | |
| 53 | HSPA4 | HSP 70 Protein 4 | Anti-apoptotic | |  |
| 54 | HSPA5 | HSP 70 Protein 5 | Anti-apoptotic | |  |
| 55 | HSPB2 | HSP27 | Anti-apoptotic | |  |
| 56 | ID-1 | Inhibitor of DNA Binding 1 | Contributes to cell growth, differentiation and angiogenesis | | |
| 57 | IGF1R | Insulin like growth factor receptor 1 | Inhibitor of apoptosis | | |
| 58 | IL-6 | Interleukin 6 | Regulated cell proliferation and acute phase responses | | |
| 59 | IL-8 | Interleukin 8 | Inflammatory response | | |
| 60 | JUN | cJun | Activates gene transcription in response to cell stimulation | | |
| 61 | MAP2K4 | MKK4 | Cell cycle control and JNK activation | | |
| 62 | MAP2K7 | MKK7 | Cell cycle control and JNK activation | | |
| 63 | MAP3K5 | ASK-1 | Activation of JNK signaling | | |
| 64 | MAP10 | Jnk3 | JNK activity | |  |
| 65 | MAPK8 | Jnk1 | JNK activity | |  |
| 66 | MAPK9 | Jnk2 | JNK activity | |  |
| 67 | MCL-1 | Mcl-1 | Anti-apoptotic | |  |
| 68 | MYC | V_MYC Avian Myelocytomatosis viral oncogene homolog | Regulation of cell proliferation and cell cycle progression | | |
| 69 | NBN | Nibrin | Double strand break repair | | |
| 70 | NGRF | Nerve growth factor receptor | Induction of apoptosis | | |
| 71 | NOL3 | Nucleolar protein 3 | Anti-apoptotic | |  |
| 72 | PDCD8 | AIF | Induction of apoptosis | | |
| 73 | PMAIP1 | NOXA | Induction of apoptosis | | |
| 74 | PTEN | PTEN | Tumour suppressor gene | | |
| 75 | RAF-1 | Raf-1 | Anti-apoptotic | |  |
| 76 | RASGRP1 | Ras granyl nucleotide-releasing protein 1 | Activates Ras | |  |
| 77 | RPA1 | Replication protein A | DNA repair | |  |
| 78 | SPAG5 | Sperm associated Ag 5 | Spindle organisation and mitosis | | |
| 79 | STAT3 | STAT3 | Inhibitor of apoptosis | | |
| 80 | TEGT | Bax inhibitor 1 | Inhibitor of apoptosis | | |
| 81 | TNF | Tumour necrosis factor | Multifunctional proinflammatory cytokine | | |
| 82 | TNFRSF10A | TNF family member 10A | Induction of apoptosis and activation of NFκB | | |
| 83 | TNFRSF10B | TNF family member 10B | Induction of apoptosis and activation of NFκB | | |
| 84 | TNFRSF10C | TNF family member 10C | Inhibitor of TRAIL-mediated apoptosis | | |
| 85 | TNFRSF10D | TRAIL R4 | Inhibition of TRAIL-induced apoptosis and activation of NFκB | | |
| 86 | TNFRSF11B | TNF family member 11B | Induction of apoptosis | | |
| 87 | TNFRSF1B | TNF family member 1B | Induction of apoptosis | | |
| 88 | TNFSF10 | TRAIL | Induction of apoptosis | | |
| 89 | TP53 | p53 | Induction of apoptosis. Regulates target genes that induce cell cycle arrest, apoptosis, senescence, DNA repair | | |
| 90 | TP73 | p73 | Mismatch repair, DNA damage response | | |
| 91 | TRADD | TNF receptor 1-associated death domain protein | Induction of apoptosis and NFκB activity | | |
| 92 | TRAF-1 | TNF receptor factor 1 | Induction and regulation of apoptosis | | |
| 93 | TRAF-2 | TNF receptor factor 2 | Induction and regulation of apoptosis | | |
| 94 | UXT | Uboquoustly expressed transcript | Microtubule organisation and marker of NFκB activity | | |
| 95 | VEGF | Vascular endothelial growth factor | Cell proliferation | |  |
